# The Decoding of the Human Spirit: A Synergy of Spirituality and Character Strengths Toward Wholeness

**DOI:** 10.3389/fpsyg.2020.02040

**Published:** 2020-09-04

**Authors:** Ryan M. Niemiec, Pninit Russo-Netzer, Kenneth I. Pargament

**Affiliations:** ^1^VIA Institute on Character, Cincinnati, OH, United States; ^2^Department of Counseling and Human Development, Achva Academic College, University of Haifa, Haifa, Israel; ^3^Department of Psychology, Bowling Green State University, Bowling Green, KY, United States

**Keywords:** spirituality, character strengths, wholeness, signature strengths, VIA classification, sacred

## Abstract

Little attention has been given to the integral relationship between character strengths and spirituality (the search for or communing with the sacred to derive meaning and purpose). The science of character strengths has surged in recent years with hundreds of studies, yet with minimal attention to spirituality or the literature thereof. At the same time, the science of spirituality has steadily unfolded over the last few decades and has offered only occasional attention to select strengths of character (e.g., humility, love, and forgiveness) or the universal typology of the VIA classification of character strengths and virtues. In this exploration, we argue that there is a robust synergy of these sciences and practices revealing that spirituality is vitally concerned with promoting character strengths. At the same time, character strengths can enhance and deepen spiritual practices, rituals, and experiences. We elaborate on how character strengths and spirituality come together in the context of the psycho-spiritual journey toward wholeness. By wholeness, we are referring to a way of being in the world that involves a life-affirming view of oneself and the world, a capacity to see and approach life with breadth and depth and the ability to organize the life journey into a cohesive whole. We further discuss six levels by which spirituality can be integrated within the VIA Classification, including a meta-perspective in which wholeness represents a meta-strength or superordinate virtue. We frame two pathways of integration: the grounding path, in which character strengths offer tangibility and thereby deepen and enhance spirituality, and the sanctification path, in which spirituality elevates character strengths. Finally, we turn to research-based practices and examine how character strengths might facilitate and contribute to spiritual practices and, conversely, how spirituality might enhance character strength practices. Such multifaceted integration offers insight and wisdom to both areas of study and opens up new directions for psycho-spiritual research and practices to deepen and broaden our understanding of what it means to be human.

“If a man is to live, he must be all alive, body, soul, mind, heart, spirit.” – Thomas Merton

## Introduction

Spirituality is a significant and universal aspect of human experience. The specific content of spiritual belief, practice, and experience varies, but all cultures have a concept of an ultimate, transcendent, sacred, or divine force (Peterson and Seligman, 2004). Spirituality is consistently defined by scientists as the search for, or communion with, the sacred (Pargament et al., 2013b). This has become nearly a consensual definition among scientists in the study of spirituality as this definition is reflected in approximately two-thirds of studies on the topic (Kapuscinski and Masters, 2010). Embedded in this definition are three core concepts – the sacred or the transcendent (beyond the ordinary), a connection or relationship with the sacred, and the search for ultimate meaning or purpose (Mayseless and Russo-Netzer, 2017). In this way, spirituality could be both a result of meaning/purpose or the source of meaning/purpose. The word “sacred” most commonly refers to God, higher power, divinity, or qualities associated with the divine, such as transcendence, ultimacy, boundlessness, and deep connectedness. People can experience the sacred through a variety of channels, such as a sense of connection, closeness, or oneness with the transcendent, a theistic being, oneself, humanity, all living beings, or nature (Davis et al., 2015).

The term “search” refers to the process of discovering, maintaining, and at times transforming a relationship with the sacred. People can search for the sacred within traditional religious contexts as well as nontraditional contexts. Moreover, pathways to the sacred can take the form of spiritual practices, such as meditation and prayer; spiritual beliefs, such as beliefs in an afterlife or karma; spiritual relationships with family, friends, or institutions; and spiritual experiences such as mystical encounters and sacred moments (Pargament et al., 2013b). It is important to add that spirituality has demonstrated a potential to bring out both the best and the worst in human nature (e.g., Pargament, 2002). We will predominantly focus here on the brighter side of spirituality.

An extensive body of scientific research has found that spirituality plays an important role in mental well-being (e.g., Paloutzian and Park, 2013; Pargament et al., 2013a) and physical health (Koenig et al., 2012) and also serves as a protective factor in psychological adjustment to negative life experiences (e.g., Gall and Guirguis-Younger, 2013).

Character strengths are also universal (Peterson and Seligman, 2004). Character strengths are defined as positive personality traits that are core to identity, elicit positive outcomes (e.g., improved well-being, relationships, health, meaning, and achievement), and contribute to the collective good (Niemiec, 2018). Modern research from a 3-year collaboration of scientists (Peterson and Seligman, 2004) involved an investigation into common humanity and the qualities of a full and meaningful life. From the “fruits of the spirit” of Saint Aquinas (1989) to the character strengths and virtues outlined by Benjamin Franklin and King Charlemagne, major texts in virtue, theology, psychology, and related fields were reviewed. Remarkable parallels across these works – spanning ancient philosophies and each of the major world religions – were found (Dahlsgaard et al., 2005). The result of this impressive project was the VIA classification of character strengths and virtues (Peterson and Seligman, 2004), a common language of 24 positive qualities that make us most human. These 24 character strengths nest under universal virtues; for example, the character strengths of curiosity and creativity fall under the wisdom virtue, bravery, and honesty under courage, love, and social intelligence under humanity, teamwork, and fairness under justice, forgiveness and prudence under temperance, and hope and gratitude under transcendence.

Studies confirmed the existence of these character strengths among human beings across cultures, nations, and beliefs (Park et al., 2006; McGrath, 2015), including people living in some of the most remote cultures on the planet, largely disconnected from modern society (Biswas-Diener, 2006). Following the emergence of this classification of human strengths, over 700 scientific studies have been published offering further validation for this typology (VIA Institute, 2020). Considering the breadth of studies on character strengths in recent years, it is surprising how few have formally examined the VIA classification of character strengths and spirituality. A couple of exceptions are Schuurmans-Stekhoven (2011) and Berthold and Ruch (2014), discussed later.

This article will explore the integration of spirituality and character strengths and consider how spirituality serves as a unique lens through which we can view, understand, and perhaps enhance character strengths, as well as how the latest science, core concepts, and best practices in character strengths inform and deepen our understanding of spirituality and offer the potential to advance spiritual practices and experiences. To provide an integrative framework, we reflect on research from a variety of methodologies and sources such as quantitative, qualitative/phenomenological, theological, psychosociological, philosophical, and other fields, as this integration requires insight from multiple perspectives as opposed to being rooted solely in one field such as positive psychology or theology.

An important initial question might be posed: why discuss the integration of character strengths and spirituality? We offer a number of thoughts on this.

- Simply put, these areas of character strengths and spirituality are the backbone of the human experience. The science of character strengths offers a wide range of practices that can be applied to spirituality and spiritual contexts, and the science of spirituality can bring unique insights to enhance our understanding and embracing of our identity – who we are at our basic core.
- Furthermore, given that processes of spiritual change and development are evident both within and outside the boundaries of institutional religious practices and traditions and considered to be “a change in the meaning system that a person holds as a basis for self-definition, the interpretation of life, and overarching purposes and ultimate concerns” (Paloutzian, 2005, p. 334), they inherently involve the use of character strengths.
- Character strengths and spirituality sit within domains of virtue, what people hold sacred, the fulfilled life, meaning and purpose, wisdom, the pursuit of moral goodness, and the enhancement of what matters most to people such as cultivating good relationships and making a positive impact on the world. In this regard, the integration of spirituality with character strengths and virtues creates an opportunity to make these positive outcomes, aspirations, and pursuits more deliberate, conscious, and a more likely reality for individuals and groups (Sandage and Hill, 2001).
- Both spirituality and character strengths share an interest in the promotion of greater wholeness. Wholeness is a dimension of well-being that goes beyond any single spiritual attribute, character strength, or virtue. Instead, it speaks to people in their entirety (Pargament et al., 2016; Russo-Netzer, 2017b). It is also multilayered and dynamic and can manifest itself in diverse ways. Wholeness has three defining features (Pargament et al., 2016, in press). First, it involves the capacity to see and approach life with breadth and depth. As a being of breadth, the individual is singular yet also a part of a larger collective, someone with a past, present, and future, a container of good and bad, and someone who knows, experiences, acts, and relates. As a being of depth, the individual is able to see beyond ordinary material existence and address matters of what theologian Tillich (1957) called “ultimate concern.” Second, wholeness involves a life-affirming view of oneself and the world. This view is filled with hope, support, and compassion in relation to oneself, other people, the world, the sacred, and life itself. Third, wholeness involves the ability to organize the life journey into a cohesive whole. Here we are referring to the capacity to put thoughts, values, emotions, actions, and relationships into an integrated totality. This mirrors what James (1936) described as moving from a divided self to a unified self, which he explained is a central spiritual task of optimal development. This capacity for wholeness, in turn, requires several specific qualities, including an authentic guiding vision, wisdom and discernment, balance, and the ability to live with paradoxes, limitations, and complexities (Russo-Netzer, 2017b).
- Character strengths offer a pathway to improve the human condition and to foster this growth and wholeness in the psycho-spiritual journey. In the words of the virtue scholar Comte-Sponville (2001, p. 3), our best qualities are both our being and becoming:
  Virtue is a way of being, Aristotle explained, but an acquired and lasting way of being: it is what we are (and therefore what we can do), and what we are is what we have become.… it is our way of being and acting humanly … our power to act *well*.
- The integration between character strengths and spirituality ultimately offers us a grounding in everyday life in addition to a perspective that everything has the potential to be sanctified as sacred. Mindfulness scholar Kabat-Zinn (1994, p. 182) offered it this way:
  Perhaps ultimately, spiritual simply means experiencing wholeness and interconnectedness directly, a seeing that individuality and the totality are interwoven, that nothing is separate or extraneous. If you see in this way, then everything becomes spiritual in its deepest sense. Doing science is spiritual. So is washing the dishes.

This integration offers a way by which we might see, experience, live, and relate to ourselves, to others, and to the world.

## The Harmony of Spirituality and Character Strengths

### Existing Links in the VIA Classification Model

There are a number of models that have linked one or more character strengths to spirituality in an important way. For example, Koenig describes strengths and virtues, such as forgiveness, gratitude, and humility, as mediators linking spirituality and health (Koenig et al., 2012). In fact, within most models or ways of thinking about spirituality, one would be hard-pressed *not* to discover one or more character strengths as an important part of the model.

The casual observer and user of the VIA classification may not be struck by the role of spirituality that can be interpreted within it. However, a careful examination of the VIA classification reveals several levels by which spirituality is infused, explicitly and implicitly. Each is relevant to our reflections on the integration of spirituality and character strengths. We start with the most specific and broaden from there.

#### Strength Level: Single Strength

The most obvious point of integration is the direct labeling of one of the 24 character traits that are ubiquitous in human beings as the strength of spirituality. This strength is defined in the VIA classification model as knowing where one fits within the larger scheme; and having beliefs about the meaning of life that shape conduct and provide comfort (Peterson and Seligman, 2004). There are several dimensions to this strength: it can be expressed through feelings and practices relating to interconnectedness, virtue, calling, religious ritual, faith, nature, meaning in life, and purpose (Niemiec and McGrath, 2019). This level represents a concrete integration of the sacred already existing within the VIA model. However, we argue that this is merely a starting point for the other levels of integration and the wider synergy discussed in this paper.

#### Strength Level: Spiritually Oriented Strengths

There are a number of specific character strengths in the VIA classification that are embedded in the sacred literatures of the world’s major religious traditions. For example, concepts of forgiveness are mentioned 234 times in the Qur’an (Rye et al., 2000). Moreover, theologians, religious leaders, and scientists in the broader field of spirituality would agree that many character strengths in the VIA classification are clearly “spiritual” in nature. These include, but are not limited to, the character strengths of humility, gratitude, forgiveness, awe (appreciation of beauty), kindness, hope, fairness, and love (for example, Saroglou et al., 2008; Carlisle and Tsang, 2013; Davis and Hook, 2014).

#### Virtue Level: Single Virtue

The strength of spirituality is nested within the larger virtue category called transcendence. Transcendence is a term from the spiritual literature that refers to moving beyond the concrete, physical world and connecting outside oneself. The original framing for the virtue of transcendence is strengths that forge connections to the larger universe and provide meaning (Peterson and Seligman, 2004). Other strengths under the virtue category of transcendence include gratitude, hope, appreciation of beauty and excellence, and humor, although the latter has subsequently been shown scientifically to align better with other virtues such as wisdom and humanity (Ruch and Proyer, 2015).

#### Virtue Level: All Six Virtues

The specific six virtues in the VIA classification – wisdom, courage, humanity, justice, temperance, and transcendence – were derived from examining the common threads or truths across all the major world religions, as well as ancient philosophies (Dahlsgaard et al., 2005). In other words, these virtues are prominent and important spiritual pathways to the sacred found in the major world religions.

#### All 24 Character Strengths as Psycho-Spiritual Qualities

We argue that each of the 24 character strengths holds the capacity to be “spiritual,” or a psycho-spiritual quality. While some strengths are more obviously aligned with spirituality (see section Strength Level: Spiritually Oriented Strengths), those less obviously aligned not only correlate with spirituality (McGrath, 2013, Unpublished) but have been shown in studies to be particularly important to it. Take the strength of self-regulation or self-control, for example, which is not traditionally viewed as a spiritual strength (although temperance is certainly a related spiritual virtue). Studies have found substantial connections in which higher levels of spirituality or the priming of spirituality led to improvements in self-regulation (Laurin et al., 2011; Watterson and Giesler, 2012). Another example is the link between creativity and spirituality (e.g., Borooah and Devi, 2015). These less obvious spiritually related strengths have the potential to add richness, depth, and perspective to self-transcendence, spiritual expression, and development.

In a related way, Peterson and Seligman (2004) offered “morally valued” as one of the main criteria for establishing and describing each of the 24 character strengths. While they were not referring to morally valued in the spiritual or sacred context, we find their comments relevant here. They explained that some character strengths are obviously morally valued, such as love and fairness, while other strengths are less clear, such as humor. They termed such strengths as “value-added strengths,” meaning that if humor is combined with a blatant morally valued strength (e.g., kindness) then humor becomes morally valued as well. For example, a comedian who uses humor to kindly cheer up sick children at a pediatric hospital would be applying his or her strength of humor in a morally valued way.

We suggest that each of the 24 character strengths can be “spiritual” or sacred and support the individual and community along their psycho-spiritual journey. Each strength is a capacity for expressing goodness – being good, doing good for others, and expressing meaning or purpose in the world. In these ways, coupled with the summation of the preceding levels, the 24 strengths can be viewed as representing a “spiritual language,” or what we call a decoding of the human spirit.

### Additional Level: Superordinate or Master Virtue

#### Wholeness Level

Building from these levels, we hypothesize a meta approach that offers wholeness as an overarching final level. Many researchers have discussed a master strength representing a higher arching virtue by which the other strengths pass through to operate or optimally express themselves – for example, self-regulation (Baumeister and Vohs, 2004), love (Vaillant, 2008), humility (Lavelock et al., 2017), and perspective/social intelligence (practical wisdom; Schwartz and Sharpe, 2006). We offer another perspective: wholeness. Wholeness shifts our focus away from the search for one key to the life well-lived (Pargament et al., 2016; Russo-Netzer, 2017b). It embraces the need to wrestle with life in its multifaceted complexity and organize it into a unified whole. To put it colloquially, wholeness has to do with how well we put the bits and pieces of our lives together, and as such, it is an ongoing, vibrant process. Although the movement from brokenness to greater wholeness has received emphasis within religious traditions, wholeness is not the antithesis of brokenness but rather involves a changed relationship to brokenness. Indeed, to be whole we must allow ourselves to get fully involved in life, be vulnerable enough to see our brokenness, and find ways to create a new compelling unity out of the broken pieces. At the core of being human lie paradoxes and dichotomies that contain the whole of existence and encapsulate completeness. The whole life is thus marked by integrity and, as noted, several defining ingredients – breadth and depth, a life-affirming orientation, and cohesiveness.

In imagining this role of master virtue, picture a wheel. Wholeness is at the center or hub of the wheel, and the 24 character strengths are the spokes directing energy toward the hub, as well as receiving energy from it. Wholeness lends unity to all 24 character strengths.

### A Spiritual Journey Model Integrating Character Strengths and Spirituality

**Figure 1:**
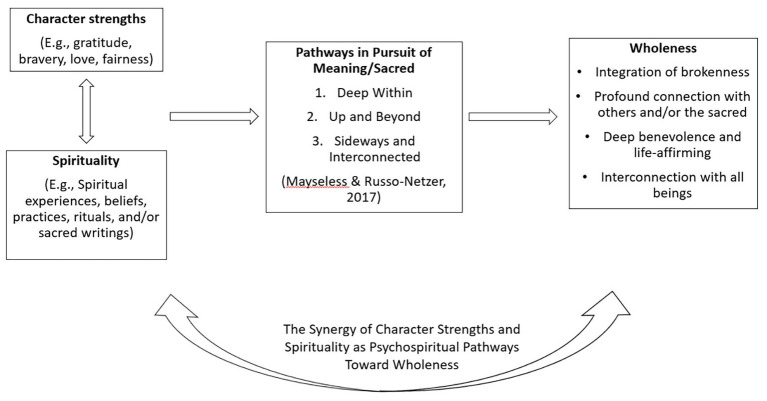
Heuristic model for the spiritual journey showing the synergy of character strengths and spirituality toward greater wholeness.

The spiritual journey is nonlinear, has no final end point, involves conscious and unconscious actions, and (at its best) is morally driven/character driven (Russo-Netzer, 2016, 2017a; Russo-Netzer and Mayseless, 2016; Mayseless and Russo-Netzer, 2017). It is directed toward a relationship with what is perceived as sacred. Figure 1 shows elements of a model of the spiritual journey through character strengths as a force for wholeness. This model incorporates the three-dimensional developmental elements of Mayseless and Russo-Netzer (2017), which are rooted in cross-cultural, spiritual, and religious literature. In brief, they argue that spiritual growth occurs across three spatial facets: deep within, up and beyond, and sideways and interconnected. These developmental elements are the “connective tissue” for the meaningful expression of character strengths and spirituality. For example, over time the individual explores, engages with, pursues, and experiences character strengths with the sacred leading toward greater wholeness. This exploring and engagement occurs as the individual (a) uses character strengths (e.g., perspective, judgment) to reach *deep within*, carefully listening to and connecting with his or her authentic self, discovering inner harmony; (b) uses character strengths (e.g., gratitude, hope) to reach *up and beyond* as he or she transcends the self and deepens his or her connection with divine or sacred presence and sees things more clearly through the lens of character strengths, such as kindness/compassion, wisdom, and awe or appreciation of beauty; (c) uses character strengths (e.g., humility, social intelligence, love) to reach *sideways and connect* with others, including all living beings and to see the interconnectedness therein with humankind and the universe.

As can be seen in our proposed heuristic model, this connective tissue catalyzed by spirituality and character strengths brings people to authentically face their suffering, challenges, and brokenness as an essential and inherent part of a full life, to connect deeply with others, and to reach up to a greater sacred presence in their journey toward wholeness.

## The Reciprocal Relationships Between Spirituality and Character Strengths

We propose that there are two main ways that spirituality and character strengths become integrated and positively impact each other. We use the term *path* or *pathway* in a conceptual way, as opposed to using it as a scientific or empirical term that definitively captures causal directions, mediating or moderating effects. To elucidate these “pathways,” we start with either spirituality or character strengths (whichever is the focal point of a research study or the best practice being primarily focused on) and then consider how it is enhanced by the other construct.

We have named the two pathways based on the dynamics we perceive to be occurring within each integration of constructs. First, we consider how character strengths can support, guide, and enhance spirituality – this process will be referred to as *the grounding path*. Then we examine the reverse direction. The application and use of spirituality to support and enhance character strengths will be referred to as *the sanctification path*. Each of these pathways is hypothesized as leading to greater wholeness. Below, we offer explanations and examples for each of these paths of integration.

### The Grounding Path: Character Strengths → Spirituality → Wholeness

In the grounding path of integration, character strengths enhance spirituality. Through this path, spirituality can become more tangible, accessible, layered, and filled with greater meaning and substance. Imagine a spiritual practice or spiritual experience devoid of love, kindness and compassion, forgiveness, humility, fairness, judgment, and critical thinking, and hope. The grounding path of integration helps deepen the awareness, expression, and meaning of spirituality through everyday experience of CS. As character strengths are ubiquitous qualities in all human beings, across cultures, nations, and beliefs (Biswas-Diener, 2006; Park et al., 2006; McGrath, 2015), the integration of character strengths into expressions of spirituality provides a way to “universalize” this dimension of human experience. The critical role of character strengths in spirituality was highlighted by Schuurmans-Stekhoven (2011) who found that well-being is more strongly associated with character strengths than spirituality, and that spirituality is related to character strengths more strongly than to well-being. Multivariate analyses showed that character strengths account for the entire positive effect of the relationship between spirituality and well-being, and argued that character strengths might be the best explanation for why spirituality has positive effects.

Any of the 24 character strengths can serve as a pathway in the seeking, dwelling, and/or maintaining of the sacred. They enable an individual to take sacred moments and experiences to a deeper level, such as when a person uses her bravery to face the challenges of being vulnerable with another person or who uses her perseverance to press forward with her spiritual practice even though many obstacles are getting in the way. One can see the potential that the grounding path could have for the person who seeks spirituality or adheres to a set of religious beliefs but is lost in a world of addictive behavior in which self-kindness, perspective, perseverance, and other character strengths are being woefully under-utilized; these strengths and others hold the potential to enhance their spirituality. See Table 1 for examples of character strengths and how each can enhance spirituality; but note that any particular strength can serve many purposes and be applied across various areas of spirituality. The areas of spirituality offered include rituals, practices, experiences, and beliefs (Hood et al., 2018).

**Table 1:** Examples of integrating character strengths into different areas of spirituality within the grounding path.

Character strength	Area of spirituality	Example of integration
Curiosity	Beliefs	Exploring and questioning the meaning of life and nature of existence.
Bravery	Practices	Facing and embracing one’s brokenness, imperfections, or “dark night of the soul.”
Prudence	Rituals	Mapping out a structured plan for daily prayer at the same time each day.
Kindness	Experiences	Volunteering to help the homeless and doing so with extra compassion and mindful kindness.
Hope	Beliefs	A person’s belief that “God is good” is strengthened by her hope/optimism during difficult times.
Zest	Rituals	Participating in a spiritual service with a jolt of energy and gusto.
Gratitude	Practices	Listing three things at the end of the day that were meaningful and writing down why one is grateful for each.
Love	Experiences	A moment of connection between two people is enhanced with a loving embrace and intimate, deep listening and appreciation.
Love of learning	Beliefs	A person studies veganism in order to learn and support his beliefs about the sacredness of life and the interconnectedness of all beings.
Self-regulation	Rituals/Practices	A person’s faltering meditation practice gains traction by a new, structured discipline of commitment to practice the same time each day.
Appreciation of beauty	Experiences	A feeling of connection in nature is enhanced by the appreciation of beauty and awe in the experience.

At this point, it is important to note that the character strengths literature suggests that humans can overuse or underuse any of the 24 character strengths (Niemiec, 2019a). Research has drawn links between an imbalance among character strengths with psychopathology (Freidlin et al., 2017). For the grounding path, the addition of character strengths has the potential to create a healthy and balanced spirituality that pursues the good for oneself, others, and all beings, and yet imbalances can occur. Too much (overuse) hope may bring a person only to look at the positive side of her religion or spirituality and omit the dark sides or limitations, while too little (underuse) judgment/critical thinking about one’s spiritual beliefs can create a narrow and selfish spiritual worldview. Balancing character strengths calls for greater wholeness, including the qualities of cohesiveness, flexibility, and discernment. It has been suggested that a grounded, everyday spirituality is one that is flexible to allow exploration and inquiry, rather than rigidity, and encourages openness and pluralism (Russo-Netzer, 2017b).

The grounding pathway of integration can also be viewed through the lens of existing spiritual models and spiritual programs in which character strengths are likely present and enhance spirituality in some way. For example, in a 4-week program addressing spiritual struggles in a religious context, sessions focused on the value of virtue, the problem with perfection, growth and grace, and relapse and reconciliation (Ano et al., 2017). Multiple character strengths – although not necessarily made explicit – can be seen in each session, such as forgiveness (the focus on cultivating this strength), hope (the focus on future growth), self-regulation (a focus on seeing the limits of self-control), spirituality (the focus on pursuing grace), and perseverance (a focus on overcoming barriers), to name just a few strength pathways designed to improve spirituality. This program was successful in helping people cultivate their virtues and resist their vices.

### The Sanctification Path: Spirituality → Character Strengths → Wholeness

The other way spirituality and character strengths can become integrated is through the sanctification path. This path involves the exploration, integration, and impact of spirituality upon character strengths. Sanctification is not used in a theological sense here. Rather, it refers to the psychological process of perceiving aspects of life as manifestations of God or as containing qualities often associated with the divine, such as transcendence, boundlessness, ultimacy, and deep connectedness (Pargament and Mahoney, 2005). A growing body of research has pointed to the benefits of instilling life domains – marriage, family, the environment, strivings, moments in time, work – with deeper spiritual meaning (Powerleau et al., 2016). People are more likely to invest in, preserve, and protect sacred aspects of life. They draw on what they hold sacred as sources of strength and inspiration. They also derive greater satisfaction, purpose, and mental health benefits from sacred objects and experience.

Any of the 24 character strengths could also be imbued with spiritual significance and meaning, lending motivational power to the strength. While each character strength has been described as a capacity for thinking, feeling, and behaving (Park et al., 2004), we believe the dimension of sacred could be added in that each character strength has the capacity to be perceived as sacred. Thus, when a strength is sanctified, its sacred dimension is being tapped into and potentially expressed.

For example, one might tap into the sacred in the character strength of love in one’s relationship with one’s spouse or child, thereby enhancing the sanctity of that bond and further increasing the love. A more general example is found in spirituality exemplars, or individuals who are living their truth and modeling a life that pursues the sacred in a positive way. Such individuals might be apt to express a wider range of character strengths because of their strong spiritual approach; in many cases, their spiritual life would not only include strengths such as kindness, humility, honesty, and forgiveness but perhaps also judgment/critical thinking, curiosity, perseverance, and leadership. Hence, the power of the sacred is being tapped in these less traditionally spiritual strengths and as a result of the spiritual living. Although relatively little research from either field has focused directly on the sanctification of character strengths (e.g., Todd et al., 2014), we believe the process of sanctification could uplift or expand character strengths. Sanctification can lend the domain of character strength a larger significance or sense of purpose. Viewed through the lens of the sacred, any character strength can become broadened and deepened.

We demonstrate this integration in Table 2 using character strength and appreciation of beauty, in the context of a simple example of someone stepping outside their house into the outdoors where nature can be seen.

**Table 2:** Three responses to beauty by a person walking into a nature scene, illustrating the distinction of the sanctification path.

Mindless use of appreciation of beauty	Mindful use of appreciation of beauty	Spiritually infused appreciation of beauty
The person does not attend to her surroundings or notice the beauty around her and behaves as if blinders are on. “I am outdoors in nature.”	The person attends to her body and the surroundings: “I see the green trees and enjoy my body’s movement as I step on stones and feel the sun warming my left cheek. I enjoy the beauty of the glistening pond and the ripples in the water.”	The person attends to her body and surroundings and connects them to a larger whole: “I see the beauty of everything green and the shimmering light on the pond and the birds flying around, and yet I feel so much more. I am connected with all of it and with something so much larger than myself. This is a sacred experience. I hold the beauty close and rest in awe of the scene, feeling aligned with it. I breathe with it. I feel a sense of aliveness and connection to it all.”

It is important to add that the processes of sanctification and character strengths in turn can be cultivated within traditional or nontraditional spiritual contexts. Spiritual and religious systems, and often the leaders therein, frequently and explicitly encourage people to see character strengths as fruits of the spirit, expressions of what it means to be a good religious person, be it a good Christian, Jew, Muslim, Buddhist, or Hindu (Pargament and Mahoney, 2005). For instance, within Christianity, members often hear the verse “And now abide faith, hope, love, these three; but the greatest of these is love” (1 Corinthians 13:13). Similarly, Jews are taught: “… what is good; and what does the Lord require of you but to do justice, and to love kindness, and to walk humbly with your God?” (Micah 6:8). Sanctification can also grow out of spiritual practices, rituals, or living a spiritually focused life. Simply sitting mindfully with or savoring a character strength can instill it with deeper spiritual value (Bryant and Veroff, 2007).

A focus on spirituality through spiritual practices seems to be linked with greater expression of character strengths. Berthold and Ruch (2014), for example, compared religious people who practice their religion, religious people who do not practice their religion, and people who are not religious. The group that practiced their religion reported a more meaningful life and scored higher on the strengths of kindness, love, hope, forgiveness, and spirituality compared with the other groups.

## Practical Implications: the Synergy of Spirituality and Character Strengths

This section offers a dual integrative approach, first highlighting evidence-based practices from the field of spirituality and how they are or might be enhanced by character strengths (the grounding path of integration). Then, we turn to the literature on character strength interventions and illustrate how spirituality can serve as an important lens or enhancer of strengths (the sanctification path of integration).

### Practices for the Grounding Path

There are a number of traditional or nontraditional spiritual practices that could serve as the backbone for the discussion here, such as types of prayer, meditation, sacred readings, exposure to nature (e.g., forest bathing), exposure to the creative arts and humanities, and a variety of rituals. Below we sample five spiritual practices that have been linked with positive outcomes (e.g., well-being). We then discuss how character strengths are already an intricate active ingredient within that practice and/or how they *could* be woven into each practice to enhance or support it.

#### Develop a Lens for the Sacred

This activity involves developing a more finely tuned mindset, or lens, through which one perceives and discovers the sacred (Powerleau et al., 2016). There are a number of avenues and successful pathways for cultivating this lens, such as creating space and time to explore sacred moments (Goldstein, 2007; Pargament et al., 2014), synchronicity experiences (Russo-Netzer and Icekson, 2020), taking a personal striving approach that links with spiritual goals or ultimate concerns (Schnitker and Emmons, 2013), and mantra use (Wachholtz and Pargament, 2005). Ultimately, this practice is about becoming a good spiritual explorer. The character strength of curiosity can be deliberately deployed in this seeking, perceiving, and exploring of what might be or is sacred to oneself. Curiosity facilitates the openness of asking exploratory questions to ponder upon oneself or discuss with others, while the strength of judgment/critical thinking can help discern healthy and harmful spirituality (Pargament, 1997; Magyar-Russell and Griffith, 2016). Other wisdom-oriented character strengths such as perspective encourage the individual to reflect on past experiences of the sacred. Similarly, creativity can catalyze brainstorming future approaches to facilitate a closer connection with the sacred.

#### Cultivate Sacred Moments

Character strengths not only have a role in developing a spiritual lens but also in the active dwelling in or experience of spiritual moments. Empirical studies have shown that the experience of sacred moments in life is associated with a number of mental health benefits, including greater meaning, purpose, and life satisfaction (Pargament et al., 2014). Building on this literature, providers have begun to create and evaluate programs that cultivate sacred moments, and character strengths can be important elements of the path. For example, McCorkle (2005) developed a 10-week manualized intervention to increase perception of sacredness in life through didactic material, discussion, and meditation. Each week focused on the sacredness embodied in a different aspect of life, including various attributes related to character strengths, such as gratitude, giving and receiving gifts, kindness to oneself, and meaning and purpose. They evaluated the effectiveness of the program with clients dealing with social anxiety. Qualitative data indicated that the program was effective in enhancing the sense of sacredness, which, in turn, fostered greater wholeness by expanding attentional focus, interrupting maladaptive thinking, and shifting behaviors that maintain social anxiety.

Similarly, Goldstein (2007, p. 1003) developed a 3-week mindfulness intervention to help people become “aware of the sacred qualities arising from moment to moment.” The program was tied to several benefits: greater spiritual well-being, greater psychological well-being, lower levels of perceived stress and greater daily spiritual experiences. In addition, focusing on sacred moments allowed participants greater access to both positive and negative emotions. Thus, the intervention appeared to encourage more wholeness by broadening and deepening emotional experience. Character strengths, which can be made explicit in cultivating sacred moments, can expand the range of possible experiences for the individual and can also play a role in grounding the person in virtuous behavior.

#### Learn From Your Spiritual Role Models

Positive influencers, role models, or exemplars are important for many facets of life and are critical enablers of many character strengths (Peterson and Seligman, 2004). Spiritual models are defined as personal or prominent figures in one’s life who function as exemplars of spiritual qualities for the observer (Oman et al., 2012). The importance of spiritual models and/or teachers as exemplars of spiritual development and change is evident in all spiritual and religious traditions (Oman and Thoresen, 2003). Interventions involving learning from spiritual role models have been shown to positively influence nonmaterialistic aspirations and self-efficacy for learning (Oman et al., 2007). For this practice, an important first step is to name the positive model or exemplar and describe how this person has been a positive influence and what has been learned from her. We propose character strengths as a valuable addition to this practice. Individuals could be encouraged to explore how character strengths influence this person and catalyze her positive and moral behavior, with questions such as, which character strengths do you appreciate most about this spiritual figure? How do they express these strengths in their actions? If you have had direct contact with this person, what character strengths do you suppose she saw in you? The questions about character strengths bring the spiritual model down-to-earth and serve as a reminder of their humanity as well as the common humanity shared with the observer. This offers an opportunity for enhanced self-efficacy as the observer is empowered to copy the character strengths of the role model in their own way.

#### Find Your Calling or Purpose in Life

Pargament (2007, p. 218) delineated a variety of psycho-spiritual assessment probes designed to help clients discover the deeper purpose in their lives. These questions include: “What are you striving for in your life? Why is it important that you are here in this world? What legacy would you like to leave behind in your life? To what or whom are you most devoted?” In order to bring character strengths into this intervention, we propose individuals be shown the list of character strengths and definitions and explore additional questions: think of a time when you pursued something particularly meaningful; which character strengths were you using most strongly? What are your “purpose-oriented” character strengths, those strengths that give you a deep sense of purpose when you use them? Which character strengths are important as part of your life legacy?

Character strengths have been found to boost calling and purpose in life. For example, in one study of the workplace, those employees who used four or more of their signature strengths at work had significantly higher levels of viewing work as a calling (Harzer and Ruch, 2012). Other studies have found certain character strengths, on average, correlate consistently highly with purpose in life – having clear goals in life and having a sense of directedness as well as holding beliefs that give life purpose. Five character strengths – curiosity, perseverance, zest, hope, and self-regulation – are among the strongest correlates of purpose in life across different studies, while a second grouping of strengths shows significant correlations with purpose, though not as strong as the first group – love, honesty, bravery, perspective, love of learning, and creativity (Harzer, 2016). These findings point to another pathway for boosting purpose and calling in life: an individual can directly target one or more of these character strengths – especially those in the first grouping – as a route toward purpose.

#### Cultivate Deep Meaning in Life

Theorists and researchers have delineated three main types of meaning: coherence, significance, and purpose (George and Park, 2016; Martela and Steger, 2016). As we focused on purpose earlier, we’ll discuss the other two areas in this practice.

Coherence is the reflection-oriented level of meaning. It is about making sense of one’s life and considering how everything fits together when considering oneself and the universe. Character strengths can be used to enhance coherence. Examples include tapping into the strength of perspective to step back and take a wider view of life so that one does not get lost in the downpour of details and stressors; using judgment/critical thinking to analyze one’s beliefs about the world and the people in it; and enlisting curiosity to question and explore life meaning and sense-making as a greater whole.

Significance is the feeling-oriented level of meaning. It involves feeling that one matters and that life matters, not only sensing and knowing the value of life but feeling that appreciation for oneself, others, and the world in a deep way. For significance, the heart-based character strengths are likely to be of central importance. An individual might consider situations in which they have deeply expressed their strengths of love, gratitude, kindness, and forgiveness and then reflect on how they have used these strengths strongly in a positive way in one of their closest relationships and how they have contributed to their sense of significance and validation.

### Practices for the Sanctification Path

We present a sampling of five character strengths practices that have been closely tied to positive outcomes (e.g., happiness). We then discuss how spirituality can be woven into these practices to amplify, widen or support these practices.

#### Strengths-Spotting Practices

The spotting of character strengths in others is one of the most common practices for recognizing, understanding, and expressing character strengths and for drawing links between abstract positive constructs and concrete behaviors. The main elements of the strengths-spotting process involve labeling the character strengths that are observed in an individual and offering an explanation, rationale, or behavioral evidence for each strength to that person (Niemiec, 2018). Research in the education context has shown that teachers’ use of strengths-spotting facilitates positive student outcomes, such as positive affect, classroom engagement, and needs satisfaction (Quinlan et al., 2019). One way to bring spirituality into this process is to weave in “spirituality-spotting.” The strengths-spotter can actively look for instances in which an individual manifests his spirituality, expresses deep meaning in life, or appears to be engaging with the sacred. The observer then offers this feedback to the person explaining what she observed. This is likely to generate new insights for the receiver.

#### Character Strengths Appreciation

Strengths-spotting can be taken to the next level by adding in an appreciation component. Appreciation is one of the main functions of character strengths and involves expressions to other people of how important or of value they are for their strengths expression – it is a valuing of who they are at their core (Niemiec, 2019b). Research has found that couples who recognize and appreciate the character strengths of their partner have higher relationship satisfaction, needs satisfaction, and relationship commitment (Kashdan et al., 2017).

As an intervention for a couple (or a friendship or other close relationship), the individuals might share examples of stories in which they saw the other person use character strengths and express appreciation to them for each of those strengths (Niemiec, 2018). This could be bolstered by encouraging the couple to reflect on the sacredness of the sharing experience; namely, how it was special, particularly intimate, or holy for them.

#### Target any Character Strength

Research has found that personality traits, and thereby character strengths, are malleable and can be impacted by deliberate interventions, among other phenomena (Borghans et al., 2008; Hudson and Fraley, 2015; Roberts et al., 2017). For example, randomized studies have shown that character strengths interventions can enhance the levels of strengths (Schutte and Malouf, 2018). Individuals interested in bolstering their bravery, perseverance, gratitude, or hope can set that strength as their target and engage in attentional, volitional, and behavioral practices to build it up. Each strength has tailored interventions (see Niemiec, 2018), such as recounting funny things to boost humor (Gander et al., 2013), counting blessings to boost gratitude (Seligman et al., 2005), or engaging in divergent thinking to build creativity (Scott et al., 2004).

After the individual does an intervention with any strength, they can then infuse the strength with the sacred. The person might sanctify the strength mentally by seeing it as part of their spirit, or sanctify it by connecting it with a special object, imbuing the symbolic object with sacred qualities in the quiet space of meditation, prayer, and appreciation (e.g., Goldstein, 2007). This process can highlight the value and importance of the strength for one’s life and for the benefit of others (Niemiec, 2014).

#### Mental Subtraction

One of the most poignant and visceral character strength activities is a well-being boosting activity involving mental subtraction (Koo et al., 2008; Ang et al., 2015). This task is referred to as “subtract a signature strength” (Niemiec, 2018). The activity invites individuals to imagine their life for 1 month without being able to use one of their signature strengths; they notice how they would be impacted and then describe their emotional experience. Common reactions include feeling lost, panicky, de-energized, bereft, and useless. This highlights the importance of one’s highest traits of character in daily life.

A natural fit here would be the addition of participants reflecting on meaning and the sacred. Following the mental subtraction, participants would be asked: what does your reaction say about what you hold sacred or what matters most to you? How does this signature strength you chose help you create and express deep meaning and value in your life? How might this strength be sacred for you?

#### Positive Reappraisal

Reframing, or positive reappraisal, is an intervention in which individuals mindfully reframe a stressful situation, event, or perception of a person as benign, valuable, or beneficial (Folkman, 1997; Garland et al., 2009). This activity can yield a more complete, honest, and balanced perspective for the situation. Character strengths are injected into the reappraisal and help reframe the problem or person in more constructive ways (e.g., stubbornness can be seen as a reflection of perseverance; inattentiveness can be a feature of curiosity; and hyperactivity can be an expression of zest; Niemiec, 2018).

To catalyze or reinforce a positive reappraisal, participants are encouraged to explore what they learned from the stressful event or how they grew or improved as a result of the problem. Spirituality has a substantive role here. The exploration can be stimulated by a number of questions: how did this problem or conflict contribute to a sense of meaning or sacredness for you? Might you discover the sacred not only within the good but also within your troubles and challenges? Could this situation be reframed as an opportunity for spiritual growth? What did you learn today that has taught you something about what it means to be you? Benevolent spiritual reappraisals have been associated with positive outcomes among hospice caregivers (Mickley et al., 1998). This meaning-loaded exploration also contributes to reappraisals of people who have offended someone in some way. These involve seeing the complex humanity of the person, as a being who has imperfections and flaws and is in need of positive growth and transformation (Witvliet et al., 2010).

## Conclusion and Future Directions

The literature on character strengths and spirituality share a concern with human functioning at its best. The fundamental human yearning to make sense of the world around us, to transcend our transient existence, to discover our unique authentic potential and calling, to seek out a relationship with something larger than our limited selves may manifest itself and be conceptualized rather differently through the prisms of spirituality and character strengths but reflect a similar core essence. Although these areas of study have operated to some extent within different silos, we have maintained that there are important theoretical connections, potential meeting points, and synergies between these two domains. We suggested two paths – the grounding path and the sanctification path – through which character strengths and spirituality can come together and facilitate each other. We then presented examples of practices within each established domain that can be enhanced by the integration of character strengths or spirituality.

Such multifaceted integration offers insight and wisdom to both areas of study and opens up new directions for psycho-spiritual research that might further explore how these constructs relate to each other, add practical value to one another, and together contribute to greater human wholeness.

Another robust area of research involves the exploration of individual differences in the experience and manifestation of character strengths and spirituality across the life span and among different cultures and populations. How might the integration of character strengths and spirituality express itself in children, adolescents, at each stage of adulthood, among religious and non-religious, and among those from Eastern, Western, and indigenous cultures? The heuristic model we have presented holds important practical implications for educators, counselors, chaplains, religious leaders, and policy makers. Such a model could be used to catalyze interventions and programs across populations and sectors.

This model can be examined more closely. One angle is through the potential master virtue of wholeness. Qualitative studies could shed important light on how people define and experience wholeness as well as the pathways they take and challenges they encounter in their efforts to realize greater integration in their lives. Empirical studies could develop measures to assess wholeness, such as the Edinger-Schons (2019) measure of oneness beliefs as they relate to life satisfaction. Research could also test the relationships between the 24 character strengths and wholeness with variables relating to growth and well-being. In this vein, Riley et al. (2017) found that several wholeness indicators (e.g., compassion for others, optimism, presence of meaning, a collaborative relationship with God, religious commitment) were linked with measures of growth. Other studies could explicate the points of connection between wholeness and character strengths.

Continuing the advancement of the thoughtful integration of character strengths and spirituality, we believe, offers exciting new directions for what it means to be human and the cultivation of greater wholeness. Exploring new horizons for research and practice may provide a fertile ground for a deeper understanding and cultivation of human flourishing, growth, and a life worth living.

## Data Availability Statement

The original contributions presented in the study are included in the article/supplementary material, further inquiries can be directed to the corresponding author.

## Author Contributions

Original outline and draft provided by lead author and substantive edits, additions, subtractions, insights, rearrangements, and contributions were made by all three authors. All authors contributed to the article and approved the submitted version.

### Conflict of Interest

The authors declare that the research was conducted in the absence of any commercial or financial relationships that could be construed as a potential conflict of interest.

The reviewer RB declared a past co-authorship with one of the authors RN to the handling editor.
